# Embodied Coordination and Psychotherapeutic Outcome: Beyond Direct Mappings

**DOI:** 10.3389/fpsyg.2018.01257

**Published:** 2018-07-23

**Authors:** Enara García, Ezequiel A. Di Paolo

**Affiliations:** ^1^IAS-Research Group, Department of Logic and Philosophy of Science, University of the Basque Country (UPV/EHU), San Sebastian, Spain; ^2^IKERBASQUE, Basque Foundation for Science, Bilbao, Spain; ^3^Centre for Computational Neuroscience and Robotics, University of Sussex, Brighton, United Kingdom

**Keywords:** enactivism, psychotherapy, nonverbal synchrony, embodiment, therapeutic alliance, interpersonal physiology

The study of interpersonal bodily coordination, both in laboratory and in semi-naturalistic conditions, can reveal subtle phenomena that take place during social interactions (Bernieri, 1988; Yale et al., 2003; Oullier et al., 2008; Paxton and Dale, 2017). The coordination of interpersonal variables spans a range of timescales and has been associated with longer-term cognitive and affective aspects of interpersonal interaction; e.g., conversation (Abney et al., 2014), teacher-student interaction (Bernieri et al., 1988), synchrony in psychotherapy (Koole and Tschacher, 2016), and interpersonal influences on physiology (Palumbo et al., 2017). A general question of interest concerns the kinds of causal and constitutive links between interactive and unconscious coordination and interpersonal affect/cognition (De Jaegher et al., 2010). This question is particularly relevant for studies of embodied social interaction during psychotherapy.

Although the idea of looking at the dynamics of social interaction in therapeutic contexts goes back to work started in the 1950s (Watzlawick et al., 1967), it is only in recent years that data gathering and analysis techniques have allowed for more systematic studies. The cost and effort of sustained long-term experiments in conditions that are difficult to control, however, means this exciting area of research is still very much under development. A valuable example study is the work by Ramseyer and Tschacher (2011). The authors investigate correlations between interpersonal body motion coordination and therapeutic outcome. While the former is easily measurable and occurs at the scale of seconds or less, the latter condenses a broad set of factors based on therapeutic experience and corresponds roughly to a timescale of whole sessions and longer. Taking this work as an example, we propose to briefly examine the possible explanations for these correlations across such qualitatively different measures and timescales. We also suggest that further analysis beyond direct correlations may provide relevant evidence linking coordination with affect and, thus, with therapeutic alliance, that is, with the collaborative relationship between patient and therapist aimed at overcoming patient's suffering (Bordin, 1979).

Ramseyer and Tschacher (2011) use motion energy analysis to record the amount of individual head and upper body movements in patient-therapist during the first few minutes of a session. Synchronization is measured as the average of patient's and therapist's cross-correlated movement. Both therapeutic process and therapeutic outcome are assessed using a series of questionnaires. Results on therapeutic process show that patient's reported self-efficacy and relationship quality, as well as pre-to-post outcome, correlates with non-verbal patient-therapy synchrony. Based on the results, the authors suggest that body synchrony between patient and therapist may predict relationship quality and therapeutic outcome. In a related study (Tschacher et al., 2014), the authors suggest that body movements are implicitly related with emotional processes and thus episodes of synchrony in these movements may reflect the patient-therapist resonance in emotion regulation. Thus, it may be seen as a manifestation of the therapeutic alliance, that is, the emotional bond in the therapeutic relationship that allows pursuing shared goals and overcome resistance to change.

Within the acknowledged limitations of these studies (such as the lack of qualitative assessment of movements), it seems plausible that bodily synchrony could be related with some kind of affective resonance and thus with therapeutic alliance. However, the force of such results does not always emerge from prior hypotheses regarding theorized relations between interpersonal synchrony, affect, and outcome (Salvatore, 2011; Koole and Tschacher, 2016; Kleinbub, 2017). It is sometimes assumed that intercorporeal synergies signal positive interpersonal affect, but this is not always the case, nor is positive affect always a sign of therapeutic progress. We think that a theory of embodied intersubjectivity underlying working hypotheses and result interpretation also needs to be made as explicit as possible (Galbusera and Fellin, 2014). In this context, taking an enactive standpoint could be a fruitful way to complement these analyses and the formulation of testable hypotheses. We repeat that the situation is rather general and that the work by Ramseyer and Tschacher is particularly useful for making this visible.

Embodied intersubjectivity, from an enactive perspective, is always directly or indirectly linked to processes of participatory sense-making (De Jaegher and Di Paolo, 2007), i.e., processes where the active embodied sense-making of a participant in a social interaction is influenced, oriented, enhanced, thwarted, and sometimes even co-constituted by the activities of other participants. One of the implications of this view is that *coordination breakdowns* and their *joint recovery* mark important events of shared sense-construction in an interaction; and by implication the more cognitively and affectively demanding the interactive scenario, the more significant and numerous we should expect such events to be. In support of this view, strict synchronous behavior seems to be modulated by the complexity of joint action contexts and is often less clearly manifest in more complex shared tasks (Wallot et al., 2016). For this reason, Di Paolo and De Jaegher (2012) and De Jaegher and Di Paolo (2007) claim that the relation between coordination dynamics and affect/cognition is not a direct mapping between presence or absence of synchronized movement and positive or negative rapport, emotion, and joint cognitive activity. In view of this, we propose that a more informative measure for this relation is the *quantity and quality of transitions between different states of coordination* rather than the absolute values of intercorporeal synchrony. Transitions out of and into states of coordination can indicate how participants deal with breakdowns and recoveries in their interaction indicating passages between different phases of the dyadic relationship. Indeed, the attempts to be emotionally attuned with the other is often manifested in the ongoing endeavor of following coherence in interpersonal coupling. This endeavor explains the coping with continuous changes in the relationship and thus, the participatory sense-making process by which the relationship is co-constructed. Transitions between moments and kinds of coordination, rather than the absolute amount of synchronization, may better reflect changes in the psychotherapeutic relationship.

Even though not all transitions in bodily synchrony are necessarily a sign of breakdown-recovery episodes, nor are all breakdowns manifested as bodily coordination transitions, looking also at transitions rather than only at average absolute values of synchrony, may provide good indications of moments in which habitual patterns of behavior change. For example, the moment in which patients acquire a new insight about themselves may be accompanied by a reduction of gesturing and backchanneling in the conversation.

In giving a dynamical systems account of therapeutic change, we need to distinguish between first-order and second-order changes (Gelo and Salvatore, 2016). First-order change encompasses every perturbation in the coupling in which the system remains organized around a quasi-stationary mode of functioning. Second-order change, instead, implies a reorganization of the components that lead to a shift to a qualitatively new pattern of relating, such as a rupture, a resignification of the therapeutic alliance, and so on. Unlike average amounts of synchrony, the study of transitions in coordination patterns can help to understand such first and second order changes in therapeutic relationships reflecting the relational resilience, that is, the capacity of the dyadic system to recover readily and adaptively from adversity and dispute and move from one quasi-stationary regime to another.

In short, the enactive perspective questions the notion that the relation between bodily synchrony and longer term affect and cognition is always that of a direct mapping from one domain to the other. Arguably, the amount of synchrony is not linearly correlated with therapeutic alliance (or other affective phenomena such as rapport between mothers and infants, e.g., Jaffe et al., 2001). Prolonged absolute synchrony would be counterproductive for therapeutic change, as would the almost total lack of it. We would not expect a therapist that follows or mimics the movements of the patient to be very successful. Breakdowns and destabilizations are not contingent phenomena the participants could do without; they are instead necessary for changes, particularly second-order changes, to occur (Gelo and Salvatore, 2016). We also question the idea that a moderate level of synchrony is, as such and by itself, good for therapeutic outcome because we do not think that the relation between shorter and longer timescales in participatory sense-making are those of a direct mapping. Indeed, as Paxton and Dale (2017) report, the interplay between high and low level constraints (e.g., conversation type vs. informative visual stimuli) on dyadic synchrony in conversations is not a simple addition but rather these constraints modulate each other in a context-dependent manner, giving rise to unique coordination patterns.

If moderate levels of synchrony correlate with positive outcomes in some cases, we hypothesize, this is also because those actual cases are likely to show significant transitions in coordination too. Therapy sessions with a high absolute synchrony in the first half followed by extremely low synchrony in the second, averaging a moderate level, seem unlikely to be effective. Synchrony may reflect the fact that metastability in the relationship is being sustained, but in order to explain significant changes, such as the attainment of clinical goals, we should study the susceptibilities to breakdowns and the capabilities for recovery of metastable dynamical conditions that give rise to new configurations of patterns of interacting.

We believe that in giving a compelling account of how alliance is constructed, in addition to synchrony, we should also study phenomena at different timescales along therapeutic processes, laborious though such a study may be. Regardless of the clinical approach used, therapeutic processes encompass a diversity of therapeutic phases in which different relational patterns predominate (Searles, 1961; Westerman et al., 1995; Morán et al., 2016; Orsucci et al., 2016; Rodríguez et al., 2018). There might be, for instance, an alliance building phase, an emotional support phase, a narrative phase, and so forth. We should expect that these different qualities will be manifested at the level of intercorporeal coordination patterns.

In support of this idea, Rodríguez et al. (2018) have measured therapist's and patient's EEG potentials along therapeutic sessions and found that there is more activation in the prefrontal cortex of the therapists during advanced periods of the process than at the beginning of the therapy. They suggest that emotional support is greater during the first sessions whereas reflective activities are more common in later stages. This does not mean that every therapeutic process has a prescribed development from emotional support toward a more rational configuration, but these results suggest the recruitment of different cognitive/affective capabilities in different psychotherapeutic phases. We can predict that these different enacted skills have a significant effect on coordination patterns. Indeed, a conversation-type effect (e.g., argument, cooperative/competitive conversation, or funny task) has been reported to be significant in modulating synchrony patterns (Tschacher et al., 2014; Paxton and Dale, 2017).

In addition to all this, we should consider the therapist not as a “prototype” therapist, that is, as having homogeneous behavior tendencies. It is known that in any psychotherapeutic approach there are individual differences in the intervention style which are relevant for the construction of therapeutic alliance (Ackerman and Hilsenroth, 2003; De Re et al., 2012). This variability is not to be averaged out, since it is revealing of alternative paths to therapeutic progress, with potentially different dynamical signatures. A strong alliance may be a background enabling condition for the patient to rehearse new behavioral patterns within the therapeutic relationship. However, this cannot be triggered without some confrontation by the therapist to old behavior patterns. Switches in the therapeutic role are also likely to imply a shift in coordination patterns.

To sum up, we suggest that an enactive theoretical background can be useful to frame coordination studies in psychotherapeutic dyads. We hypothesize that the relation between bodily coordination and longer timescale phenomena, such as affect and therapeutic alliance, would be better accounted for in terms of transition dynamics rather than by absolute measures of synchrony. Testing this should not be methodologically onerous. It may be possible to start by defining and recording the amount and type of synchrony transition events in existing or similar datasets. At the same time, we suggest that a more complete picture requires us to explore the relation between different timescales in the therapeutic process, that is, between different therapeutic phases and styles of interventions.

## Author contributions

EG and ED contributed to the conception and design of the paper. EG wrote the first draft of the manuscript. ED wrote relevant sections of the manuscript. Both authors contributed to manuscript revision, read and approved the submitted version.

### Conflict of interest statement

The authors declare that the research was conducted in the absence of any commercial or financial relationships that could be construed as a potential conflict of interest.
